# Increased matrix metalloproteinases expression in tuberous sclerosis complex: modulation by microRNA 146a and 147b *in vitro*

**DOI:** 10.1111/nan.12572

**Published:** 2019-07-01

**Authors:** D. W. M. Broekaart, J. van Scheppingen, J. J. Anink, L. Wierts, B. van het Hof, F. E. Jansen, W. G. Spliet, P. C. van Rijen, W. W. Kamphuis, H. E. de Vries, E. Aronica, E. A. van Vliet

**Affiliations:** ^1^ Department of (Neuro)Pathology Amsterdam Neuroscience Amsterdam UMC University of Amsterdam Amsterdam The Netherlands; ^2^ Brendinn Therapeutics Amsterdam The Netherlands; ^3^ Department of Molecular Cell Biology and Immunology Amsterdam Neuroscience Amsterdam UMC Vrije Universiteit Amsterdam Amsterdam The Netherlands; ^4^ Department of Pediatric Neurology University Medical Center Utrecht Utrecht The Netherlands; ^5^ Department of Pathology University Medical Center Utrecht Utrecht The Netherlands; ^6^ Department of Neurosurgery Rudolf Magnus Institute for Neuroscience University Medical Center Utrecht Utrecht The Netherlands; ^7^ Stichting Epilepsie Instellingen Nederland (SEIN) Heemstede The Netherlands; ^8^ Swammerdam Institute for Life Sciences Center for Neuroscience University of Amsterdam Amsterdam The Netherlands

**Keywords:** blood‐brain barrier, extracellular matrix, matrix metalloproteinases, microRNA, tuberous sclerosis complex

## Abstract

**Aim:**

Matrix metalloproteinases (MMPs) and their endogenous tissue inhibitors (TIMPs) control proteolysis within the extracellular matrix (ECM) of the brain. Dysfunction of this enzymatic system due to brain inflammation can disrupt the blood‐brain barrier (BBB) and has been implicated in the pathogenesis of epilepsy. However, this has not been extensively studied in the epileptogenic human brain.

**Methods:**

We investigated the expression and cellular localization of major MMPs (MMP2, MMP3, MMP9 and MMP14) and TIMPs (TIMP1, TIMP2, TIMP3 and TIMP4) using quantitative real‐time polymerase chain reaction (RT‐PCR) and immunohistochemistry in resected epileptogenic brain tissue from patients with tuberous sclerosis complex (TSC), a severe neurodevelopmental disorder characterized by intractable epilepsy and prominent neuroinflammation. Furthermore, we determined whether anti‐inflammatory microRNAs, miR146a and miR147b, which can regulate gene expression at the transcriptional level, could attenuate dysregulated MMP and TIMP expression in TSC tuber‐derived astroglial cultures.

**Results:**

We demonstrated higher mRNA and protein expression of MMPs and TIMPs in TSC tubers compared to control and perituberal brain tissue, particularly in dysmorphic neurons and giant cells, as well as in reactive astrocytes, which was associated with BBB dysfunction. More importantly, IL‐1β‐induced dysregulation of *MMP3*,* TIMP2*,* TIMP3 and TIMP4* could be rescued by miR146a and miR147b in tuber‐derived TSC cultures.

**Conclusions:**

This study provides evidence of dysregulation of the MMP/TIMP proteolytic system in TSC, which is associated with BBB dysfunction. As dysregulated MMP and TIMP expression can be ameliorated *in vitro* by miR146a and miR147b, these miRNAs deserve further investigation as a novel therapeutic approach.

## Introduction

Matrix metalloproteinases (MMPs) are extracellular zinc‐dependent proteases characterized by a conserved methionine residue at the active site 1. Membrane‐bound or secreted latent forms of MMPs require the removal of the propeptide domain for optimal activity. This proteolytic removal can be mediated by other, already active, MMPs or by other proteases, in particular, members of the plasminogen‐plasmin system 2, 3. The activity of MMPs can be regulated by their endogenous tissue inhibitors (TIMPs) of which four forms are present in the brain 4. All TIMPs are capable of inhibiting all MMPs, though with varying efficacy 5. MMPs are able to use as substrates, all components of the extracellular matrix (ECM) 6, as well as several intracellular components 7. Under normal physiological conditions, neuronal activity can stimulate the release of MMPs from neurons 8, 9 and MMP activity is involved in dendritic spine enlargement during hippocampal long‐term potentiation and the increase in glutamatergic neurotransmission 10. They also play an important role in neurite outgrowth, cell migration and neuroinflammation 11, 12, 13, 14, 15. Under pathological conditions, MMPs can induce blood‐brain barrier (BBB) dysfunction via degradation of the basal lamina of cerebral blood vessels and disruption of tight junctions between endothelial cells 16. Due to the accumulation of serum proteins that enter the brain, causing a pro‐inflammatory response and increased neuronal excitability, BBB dysfunction can contribute to epileptogenesis 17, 18, 19, 20.

Epilepsy is one of the major clinical symptoms in patients with tuberous sclerosis complex (TSC) 21. TSC is caused by a mutation in either the *TSC1* or *TSC2* gene, resulting in a constitutive activation of the mammalian target of rapamycin (mTOR) pathway 22, 23. This multisystem disorder affects a large range of organs including the brain 24. Brain pathology in TSC patients is associated with a complex clinical phenotype including epilepsy (often intractable to medical treatment), autism and intellectual disability 23, 25, 26. Focal malformations in cortical cytoarchitecture, also known as cortical tubers, are a pathological hallmark of TSC. These tubers are characterized by the presence of abnormal cells called dysmorphic neurons and giant cells and their localization is often associated with the presence of an epileptogenic focus in TSC patients 22, 27. Furthermore, prominent brain inflammation and BBB dysfunction are observed 23, 28, 29, 30, 31.

As MMPs may contribute to TSC pathophysiology and epileptogenesis, understanding their role could have implications for the treatment of neurological symptoms. We therefore investigated the expression and localization of MMPs and TIMPs in resected brain tissue of patients with TSC in relation to BBB dysfunction. Furthermore, we determined whether anti‐inflammatory microRNAs (miRs), miR146a and miR147b, could attenuate dysregulated MMP and TIMP expression in TSC tuber‐derived astroglial cultures. miRs, which are short non‐coding RNAs, approximately 18–23 nucleotides in length, have therapeutic potential as they are capable of regulating target gene expression at the post‐transcriptional level 32. Recently, we showed that inhibition of the pro‐inflammatory miR155 could attenuate interleukin (IL)‐1β‐induced overexpression of MMP3 in cultured human astrocytes 33. Moreover, we showed in a previous study that both miR146a and miR147b can act as negative‐feedback regulators of inflammatory responses 34, which may also affect MMP and TIMP expression. Although miR146a and miR147b do not directly target MMPs, we hypothesized that by interfering with brain inflammation using these miRNAs (which directly target pro‐inflammatory genes), we can indirectly reduce MMP expression.

## Materials and methods

### Subjects

The cases included in this study were obtained from the archives of the departments of Neuropathology of the Amsterdam UMC (University of Amsterdam, The Netherlands) and the University Medical Center Utrecht (Utrecht, The Netherlands). For immunohistochemical analyses and real‐time polymerase chain reaction (RT‐PCR), cortical tubers from, respectively, 6 and 16 TSC patients were examined from whom neocortical tuber tissue was surgically removed. Presurgical evaluation, including long‐term video‐EEG monitoring, high‐resolution MRI and neuropsychological testing was performed in order to characterize the epileptogenic zone. A group of 20 autopsy gender‐ and age‐matched control cases without a history of seizures or other neurologic diseases were also included. Clinical findings of both TSC patients and control autopsy patients can be found in Table S1. None of the TSC patients received mTOR inhibitors. The age and gender did not differ between TSC patients and autopsy controls (*P* > 0.05, Mann–Whitney *U*‐Test). Tissue was obtained and used in accordance with the Declaration of Helsinki and the Amsterdam UMC Research Code provided by the Medical Ethics Committee and the local ethical committees of the participating centres gave permission to undertake the study.

### Immunohistochemistry

Human brain tissue was fixed in 10% buffered formalin and embedded in paraffin. Paraffin‐embedded tissue was sectioned at 5 μm, mounted on precoated glass slides (Star Frost, Waldemar Knittel, Braunschweig, Germany) and processed for immunohistochemical staining. Sections were deparaffinated in xylene, rinsed in ethanol (100%, 96%, 70%) and incubated for 20 min in 0.3% hydrogen peroxide diluted in methanol. Antigen retrieval was performed using a pressure cooker in 10 mM sodium citrate, pH 6.0 at 121°C for 10 min. Slides were washed with phosphate‐buffered saline (PBS, pH 7.4) and incubated overnight with primary antibody (1:200 rabbit polyclonal IgG anti‐MMP2, Abcam, Cambridge, UK; 1:100 rabbit monoclonal IgG anti‐MMP3, Abcam; 1:100 mouse monoclonal IgG_2a_ anti‐MMP9, Millipore, Amsterdam, The Netherlands; 1:500 mouse monoclonal IgG_3_/κ anti‐MMP14, Millipore; 1:200 mouse monoclonal clone VT7 anti‐TIMP1, Agilent, Santa Clara, CA, USA; 1:100 mouse monoclonal IgG_2a_/κ anti‐TIMP2, Millipore; 1:300 mouse monoclonal IgG_1_/κ anti‐TIMP3, Millipore; 1:600 rabbit polyclonal IgG anti‐TIMP4, Abcam; 1:80 000 rabbit anti‐human albumin, DAKO/Agilent Technologies Netherlands, Amstelveen, The Netherlands; or 1:300 mouse anti‐rat CD163, AbD Serotec, Dusseldorf, Germany) in PBS at 4°C. For single labelling, staining was performed using 3,3′‐diaminobenzidine substrate solution (1:10 in 0.05 M Tris–HCl, pH 7.6; ImmunoLogic, Duiven, The Netherlands) with 0.015% H_2_O_2_. The reaction was stopped by washing with distilled water after which sections were dehydrated in alcohol and xylene and coverslipped. Negative controls were performed without primary antibody (the sections were blank).

Cell‐specific double‐labelling of MMPs and TIMPs was performed with glial fibrillary acidic protein (GFAP; 1:4000 polyclonal rabbit, DAKO, Glostrup, Denmark; or 1:4000 monoclonal mouse, Sigma, St. Louis, MO, USA), ionized calcium binding adapter molecule‐1 (IBA‐1; 1:2000 polyclonal rabbit, WAKO, Richmond, VA, USA) or CD11b/c (1:100 OX‐42; DAKO/Agilent Technologies Netherlands, Amstelveen, The Netherlands). After overnight incubation at 4°C and rinsing, sections were incubated with secondary antibodies Alexa Fluor 488 donkey anti‐mouse IgG (H+L) and Alexa Fluor 568 goat anti‐rabbit IgG (H+L) (1:200; Invitrogen, Eugene, OR, USA) for 2 h at room temperature, washed in PBS and coverslipped. Fluorescent microscopy was performed using a confocal microscope (SP8‐X, Leica, Son, The Netherlands).

For double‐labelling of MMPs and TIMPs, specific MMP and TIMP combinations were chosen based on their interaction as described in literature 35. The combinations were: MMP2/TIMP2, MMP3/TIMP1, MMP3/TIMP3, MMP9/TIMP1, MMP14/TIMP2, MMP14/TIMP4. Double‐labelling was performed using BrightVision poly‐alkaline phosphatase (AP)‐anti‐rabbit or anti‐mouse (ImmunoLogic) incubated for 30 min at room temperature and washed with PBS. AP activity was visualized with the AP substrate kit III Vector Blue (SK‐5300; Vector Laboratories Inc., Burlingame, CA, USA). For double‐labelling with the same species antibodies, sections were incubated in 10 mM sodium citrate, pH 6.0 at 121°C for 10 min in a pressure cooker after visualizing the first protein of interest to denature the first primary antibody and prevent non‐specific binding of the second primary antibody. After washing in PBS, sections were stained with a polymer‐based peroxidase immunocytochemistry detection kit (Power‐Vision Peroxidase system; ImmunoVision, Brisbane, CA, USA). Signal was detected using chromogen 3‐amino‐9‐ethylcarbazole (Sigma‐Aldrich) in 0.05‐M acetate buffer (pH 8.2). Sections were dried and coverslipped with Vectashield (Vector Laboratories Inc.).

### Evaluation of immunohistochemistry

Immunoreactivity of MMPs and TIMPs was quantified by measuring the optical density (OD) of (dysmorphic) neurons, giant cells and glia within cortical tubers of patients using ImageJ as well as of neurons and glia within the neocortex of controls and perituberal cortex of TSC patients. For each case, photographs of representative brain areas were taken at 20× and the OD of six to 12 individual cells was measured after which the background signal of the respective picture was subtracted. Afterwards, the mean OD was calculated per subject. Additionally, the relative number of positive cells (0: no; 1: single to 10%; 2: 11‐50%; 3: >50%) was evaluated as described previously 36, 37.

### TSC cell cultures

Primary TSC cell cultures were derived from surgical brain specimens obtained from two patients (age at surgery: 2.5 and 2 years; gender: male; mutation: TSC2) undergoing epilepsy surgery at the Wilhelmina Children's Hospital of the University Medical Center Utrecht (Utrecht, The Netherlands). Cell isolation was performed as described elsewhere 38, 39. Briefly, after removal of blood vessels, tissue was mechanically minced into smaller fragments and enzymatically digested by incubating at 37°C for 30 min with 2.5% trypsin (Sigma‐Aldrich). Tissue was washed with incubation medium containing Dulbecco's modified Eagle's medium (DMEM)/HAM F10 (1:1) medium (Gibco, Life Technologies, Grand Island, NY, USA), supplemented with 1% penicillin/streptomycin and 10% foetal calf serum (Gibco, Life Technologies) and triturated by passing through a 70‐μm mesh filter. The cell suspension was incubated at 37°C, 5% CO_2_ for 48 h to let cells adhere the culture flask before it was washed with PBS to remove excess of myelin and cell debris. Cultures were subsequently refreshed twice a week.

### Transfection and stimulation of cell cultures

Cells plated in poly‐L‐lysin‐coated plates were transfected with mimic pre‐miRNA for miR146a or miR147b (Applied Biosystems, Carlsbad, CA, USA). Oligonucleotides were delivered to the cells using Lipofectamine^®^ 2000 transfection reagent (Life Technologies) in a final concentration of 50 nM for a total of 24 h before the start of stimulation. Astrocyte cultures were stimulated with human recombinant (r)IL‐1β (10 ng/ml; Peprotech, Rocky Hill, NJ, USA) for 24 h. The viability of human cell cultures was not influenced by the treatment with IL‐1β (as shown before; 40). The cells were harvested after 24 h of stimulation.

### RNA isolation and RT‐PCR

For RNA isolation, cell culture or fresh brain material was homogenized in Qiazol Lysis Reagent (Qiagen Benelux, Venlo, The Netherlands). Total RNA was isolated using the miRNeasy Mini kit (Qiagen Benelux) according to the manufacturer's instructions. The concentration and purity of RNA were determined at 260/280 nm using a Nanodrop 2000 spectrophotometer (Thermo Fisher Scientific, Wilmington, DE, USA). To evaluate mRNA expression, 200–500 ng of cell culture‐derived total RNA and 2500 ng of fresh brain‐derived total RNA were reverse‐transcribed into cDNA using oligo dT primers. Expression of MMPs and TIMPs was evaluated as described previously 40 using the following primer sequences: MMP2 (forward primer ataacctggatgccgtcgt, reverse primer aggcacccttgaagaagtagc); MMP3 (forward primer ctccaaccgtgaggaaaatc, reverse primer catggaatttctcttctcatcaaa); MMP9 (forward primer gaaccaatctcaccgacagg, reverse primer gccacccgagtgtaaccata); MMP14 (forward primer gccttggactgtcaggaatg, reverse primer aggggtcactggaatgctc); TIMP1 (forward primer gggcttcaccaagacctaca, reverse primer tgcaggggatggataaacag); TIMP2 (forward primer tgcagatgtagtgatcagggc, reverse primer tctcaggccctttgaacatc); TIMP3 (forward primer gctggaggtcaacaagtacca, reverse primer cacagccccgtgtacatct) and TIMP4 (forward primer ttggtgcagagggaaagtct, reverse primer ggtactgtgtagcaggtggtga). Quantification of data was performed using the LinRegPCR program in which linear regression on the Log (fluorescence) per cycle number data is applied to determine the amplification efficiency per sample 41. The starting concentration of each specific product was divided by the geometric mean of the starting concentration of the reference genes *EF1*α and *C1orf43* and this ratio was compared between groups.

### Statistical analysis

Statistical analysis of immunohistochemistry was performed using IBM SPSS Statistics 22 (IBM Nederland, Amsterdam, The Netherlands) using the Mann–Whitney *U*‐Test. For mRNA data from cell culture and human tissue, statistical analyses were performed with GraphPad Prism software (GraphPad Software Inc., La Jolla, CA, USA) using the Mann–Whitney *U*‐Test. A *P* < 0.05 was assumed to indicate a statistical difference.

## Results

### Matrix metalloproteinase expression in control cortex and cortical tubers

**Figure 1:**
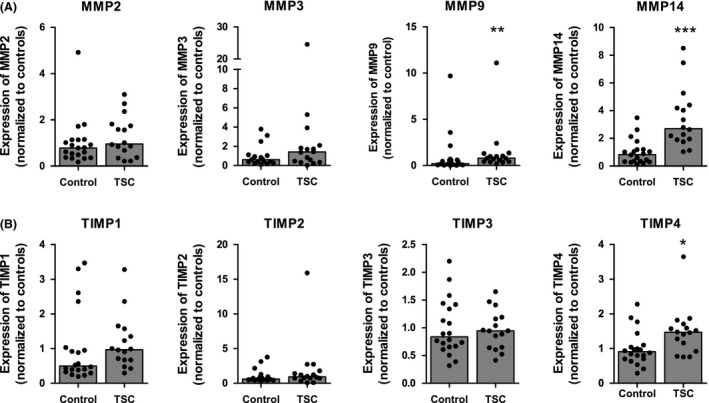
mRNA expression of matrix metalloproteinases (MMPs) and tissue inhibitor of metalloproteinase (TIMPs) in tuberous sclerosis complex (TSC) tubers. Quantitative real‐time PCR shows higher mRNA expression of *MMP9*,* MMP14* (**A**) and *TIMP4* (**B**) in cortical tubers from TSC patients compared to control tissue. mRNA expression of *MMP2*,* MMP3*,* TIMP1*,* TIMP2*,* TIMP3* and *TIMP4* did not change. *P < 0.05, **P < 0.01, ***P < 0.001; Mann–Whitney *U*‐Test.

There was higher gene expression in cortical tubers of patients with TSC *vs*. neocortical control tissue for *MMP9* (*P* < 0.01; Figure 1 **A**) and *MMP14* (*P* < 0.001; Figure 1 **A**). No significant changes in gene expression were detected for *MMP2* and *MMP3* (Figure 1 **A**).

**Figure 2:**
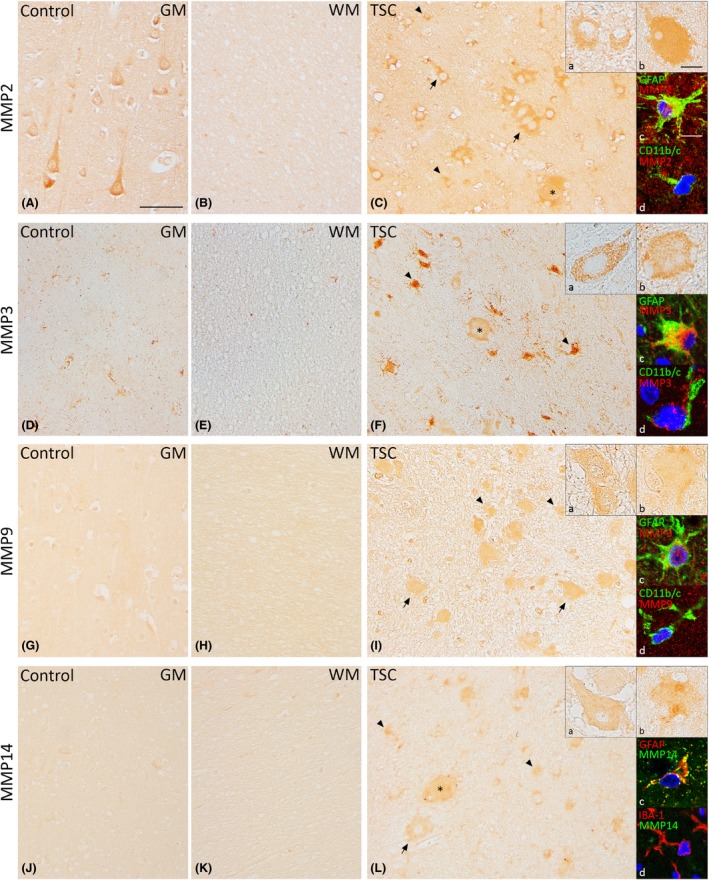
Expression of matrix metalloproteinases 2 (MMP2), MMP3, MMP9 and MMP14 in TSC tubers. Strong MMP2 expression in neurons and weak MMP2 expression in glial cells were seen in control cortex (**A**,**B**). In TSC tubers, strong MMP2 expression was observed in dysmorphic neurons, giant cells and glial cells. (**C**). MMP3 expression was not observed in neurons and weakly in glial cells of controls (**D**,**E**). Moderate expression was observed in dysmorphic neurons and giant cells, while expression was strong in glial cells (**F**). Moderate MMP9 expression was observed in neurons and no to weak expression was seen in glial cells of control cortex (**G**,**H**). In TSC tubers, dysmorphic neurons, giant cells and astrocytes showed moderate MMP9 expression (**I**). MMP14 expression was weak in neurons and not present in glial cells in controls (**J**,**K**). Moderate expression of MMP14 was observed in dysmorphic neurons, giant cells and astrocytes of TSC tubers (**L**). Insets a show magnification of dysmorphic cells, insets b show magnification of giant cells. Insets c and d in **C**,**F**,**I** and **L** depict double‐labelling of MMPs with glial fibrillary acidic protein (GFAP)‐positive cells (astrocytes) and IBA‐1 or CD11b/c‐positive cells (microglia), counterstained with 4′,6‐diamidino‐2‐phenylindole (DAPI, blue). GM, grey matter; WM, white matter; TSC, tuberous sclerosis complex. Arrows depict dysmorphic neurons, arrowheads depict glial cells and asterisks depict giant cells. Scale bar **A**–**I**: 100 μm; insets a, b: 25 μm; insets c, d: 12.5 μm.

Resected neocortical tissue from six patients with TSC was used for immunohistochemical analysis of protein expression of MMPs and TIMPs and compared to neocortical tissue from six controls. Strong MMP2 protein expression was observed in neurons, while no to weak staining was seen in glial cells of control cortex (Figure 2 **A**,**B**). In TSC tubers, strong MMP2 expression was observed in dysmorphic neurons and giant cells (Figure 2 **C**). The OD and the relative number of MMP2‐positive cells were higher in glial cells of cortical tubers compared to controls (*P* < 0.01; Table 1). Furthermore, the relative number of MMP2‐positive glial cells was higher in cortical tubers compared to perituberal cortex.

**Table 1:** Immunoreactivity analysis of matrix metalloproteinases 2 (MMP2), MMP3, MMP9 and MMP14

	(Dysmorphic) Neurons		Glia		Giant cells	
	OD	Rel. no.	OD	Rel. No.	OD	Rel. no.
MMP2						
Control	25 ± 4	3	13 ± 3	1 (1–2)	–	–
Tuber	30 ± 2	3	32 ± 3**	3*** ^,^ ^^	26 ± 3	3
Perituber	23 ± 8	3 (2–3)	23 ± 6	2 (2–3)**	–	–
MMP3						
Control	−1 ± 1	3 (1–3)	54 ± 6	1 (1–2)	–	–
Tuber	16 ± 5	2.5 (1–3)	58 ± 7	2.5 (1–3)*	10 ± 4	2.5 (1–3)
Perituber	2 ± 2	3	40 ± 7	2 (1–3)	–	–
MMP9						
Control	9 ± 2	3 (2–3)	4 ± 3	2 (1–3)	–	–
Tuber	13 ± 1	3	13 ± 2*	2 (2–3)	12 ± 2	3
Perituber	8 ± 2	3	8 ± 5	3 (1–3)	–	–
MMP14						
Control	9 ± 2	2.5 (1–3)	−1 ± 0	1	–	–
Tuber	15 ± 2	3 (2–3)	17 ± 2** ^,^ ^	2.5 (2–3)**	17 ± 5	3 (2–3)
Perituber	5 ± 3	3	5 ± 2	3**	–	–

The background‐corrected optical density (OD) is given as mean ± SE of the mean. The relative number (Rel. no.) of positive cells is given as median (minimum–maximum) and is defined as follows: 0: no; 1: single to 10%; 2: 11–50%; 3: >50%. Different compared to control, *P < 0.05, **P < 0.01, ***P < 0.001. Different compared to tuberous sclerosis complex perituber, ^^^ *P* < 0.05, ^^^^ *P* < 0.01.

In control tissue, MMP3 protein expression was not observed in neurons and moderately to strongly expressed in the present glial cells (Figure 2 **D**,**E**). In TSC tubers, however, the relative number of MMP3‐positive glia was higher compared to controls (*P* < 0.05; Table 1). Additionally, moderate expression of MMP3 was observed in dysmorphic neurons and giant cells (Figure 2 **F**).

Moderate MMP9 protein expression was observed in neurons while no to weak expression was observed in glial cells of control tissue (Figure 2 **G**,**H**). TSC tubers had moderate expression of MMP9 in dysmorphic neurons and giant cells (Figure 2 **I**). The OD of MMP9‐positive cells was higher in glia cells in TSC tubers compared to controls (Table 1).

MMP14 protein expression was weak in neurons and not present in glial cells of control tissue (Figure 2 **J**,**K**). MMP14 expression in TSC tubers was moderate in dysmorphic neurons and giant cells (Figure 2 **L**). The OD in TSC tubers was higher compared to both controls (*P* < 0.01) and perituberal cortex (*P* < 0.05; Table 1). The relative number of MMP14‐positive glia was higher in both tuber and perituberal cortex compared to control tissue (*P* < 0.01; Table 1).

Double‐labelling experiments showed that all MMPs co‐localized with the astrocytic marker GFAP (Figure 2 insets c) and that MMP2 and MMP3 also co‐localized with the microglial marker CD11b/c (Figure 2 insets d).

### Tissue inhibitor of metalloproteinase expression in control cortex and in cortical tubers

There was higher gene expression in cortical tuber of patients with TSC *vs*. neocortical control tissue for *TIMP4* (*P* < 0.05; Figure 1 **B**), while no significant changes in gene expression were detected for *TIMP1*,* TIMP2* and *TIMP3* (Figure 1 **B**).

**Figure 3:**
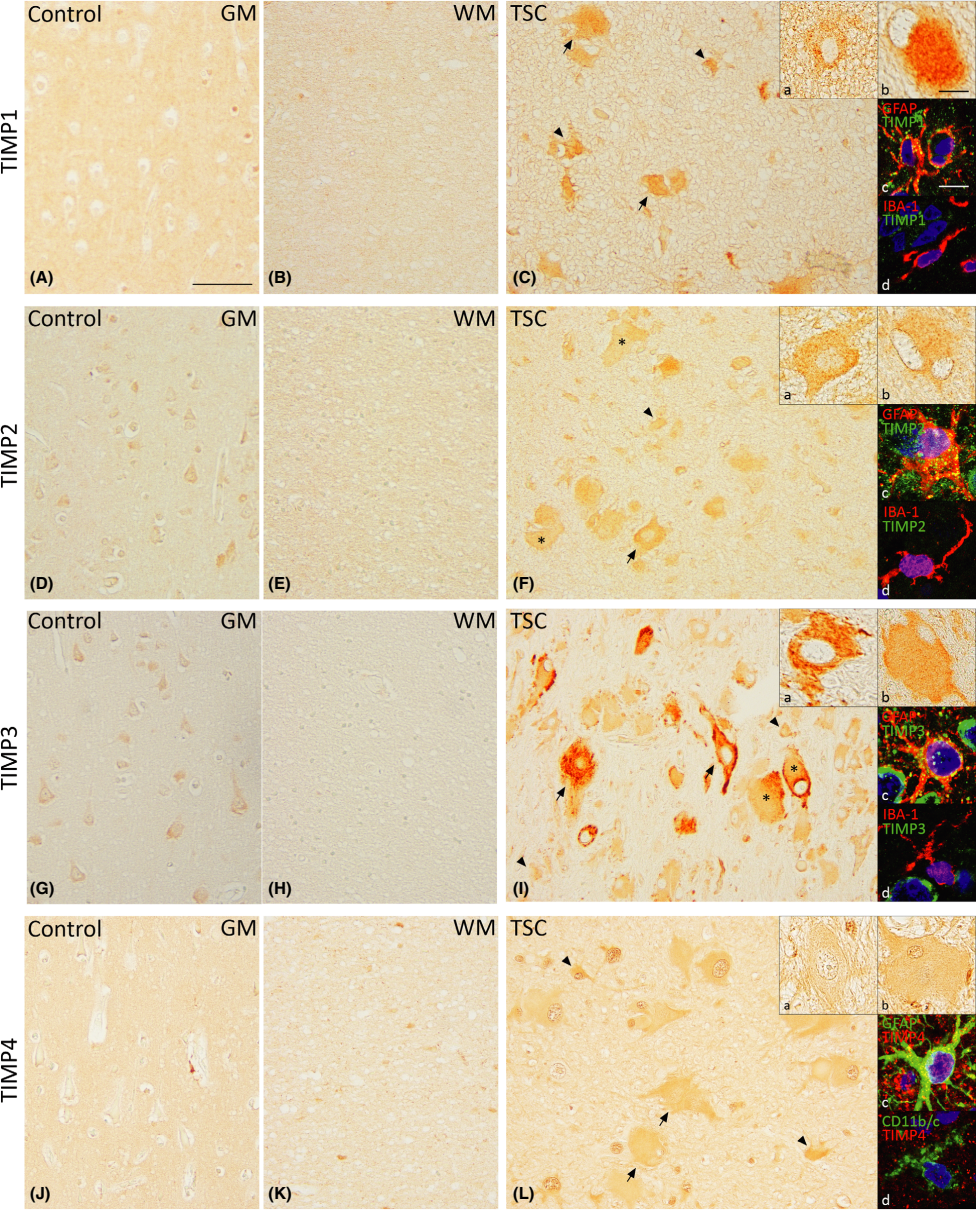
Expression of tissue inhibitor of metalloproteinase 1 (TIMP1), TIMP2, TIMP3 and TIMP4 in TSC tubers. Weak TIMP1 expression in neurons and moderate expression in glial cells were observed in control cortex (**A**,**B**). In TSC, TIMP1 was moderately expressed in dysmorphic neurons, giant cells and astrocytes (**C**). Moderate expression of TIMP2 in neurons and weak expression in glial cells were seen in control tissue (**D**,**E**). In TSC, dysmorphic neurons and giant cells showed strong TIMP2 expression while astrocytes showed moderate TIMP2 expression (**F**). TIMP3 was moderately expressed in neurons only in controls (**G**,**H**). Strong TIMP3 expression was seen in dysmorphic neurons, giant cells and astrocytes in TSC tubers (**I**). In control cortex, TIMP4 expression was moderate in glial cells only (**J**,**K**). Strong TIMP4 expression was seen in dysmorphic neurons, giant cells and astrocytes of TSC tubers (**L**). Insets a show magnification of dysmorphic cells, insets b show magnification of giant cells. Insets c and d in **C**,**F**,**I** and **L** depict double‐labelling of matrix metalloproteinases (MMPs) with glial fibrillary acidic protein (GFAP)‐positive cells (astrocytes) and IBA‐1 or CD11b/c‐positive cells (microglia), counterstained with 4′,6‐diamidino‐2‐phenylindole (DAPI, blue). GM, grey matter; WM, white matter; TSC, tuberous sclerosis complex. Arrows depict dysmorphic neurons, arrowheads depict glial cells and asterisks depict giant cells. Scale bar **A**–**I**: 100 μm; insets a, b: 25 μm; insets c, d: 12.5 μm.

In control tissue, weak TIMP1 protein expression in neurons and moderate TIMP1 expression was observed in glial cells (Figure 3 **A**,**B**). In cortical tubers, moderate expression of TIMP1 was observed in dysmorphic neurons, giant cells and astrocytes (Figure 3 **C**). The OD of TIMP1‐positive glia in tubers was higher compared to perituberal cortex (*P* < 0.01; Table 2). The relative number of TIMP1‐positive glia was higher in TSC tubers compared to controls (*P* < 0.05, Table 2).

**Table 2:** Immunoreactivity analysis of tissue inhibitor of metalloproteinase 1 (TIMP1), TIMP2, TIMP3 and TIMP4

	(Dysmorphic) Neurons		Glia		Giant cells	
	OD	Rel. no.	OD	Rel. no.	OD	Rel. no.
TIMP1						
Control	4 ± 6	2 (1–3)	27 ± 6	1.5 (1–2)	–	–
Tuber	25 ± 5	2.5 (1–3)	36 ± 5^^	2.5 (2–3)*	14 ± 3	2.5 (1–3)
Perituber	0 ± 4	3 (2–3)	3 ± 5*	1 (1–3)	–	–
TIMP2						
Control	4 ± 6	3 (2–3)	27 ± 6	1	–	–
Tuber	33 ± 4	3	39 ± 5	3*** ^,^ ^^	29 ± 4	3
Perituber	21 ± 2	3	24 ± 4	2**	–	–
TIMP3						
Control	24 ± 2	2.5 (2–3)	8 ± 1	1	–	–
Tuber	93 ± 11	3	58 ± 12**	2.5 (2–3)**	48 ± 11	3
Perituber	44 ± 6	3	32 ± 9*	2.5 (2–3)**	–	–
TIMP4						
Control	1 ± 4	3 (2–3)	64 ± 7	1 (1–2)	–	–
Tuber	50 ± 13	3 (2–3)	64 ± 6	3 (2–3)** ^,^ ^	38 ± 8	3 (2–3)
Perituber	10 ± 5	3	53 ± 8	2 (2–3)**	–	–

The background‐corrected optical density (OD) is given as mean ± SE of the mean. The relative number (Rel. no.) of positive cells is given as median (minimum–maximum) and is defined as follows: 0: no; 1: single to 10%; 2: 11–50%; 3: >50%. Different compared to control, *P < 0.05, **P < 0.01, ***P < 0.001. Different compared to tuberous sclerosis complex perituber, ^^^ *P* < 0.05, ^^^^ *P* < 0.01.

Moderate protein expression of TIMP2 was observed in neurons, while weak expression was seen in glial cells of control brain tissue (Figure 3 **D**,**E**). In cortical tubers, dysmorphic neurons and giant cells showed strong expression of TIMP2 (Figure 3 **F**). The relative number of TIMP2‐positive glia was higher in both tubers (*P* < 0.001) and perituber (*P* < 0.01) compared to controls, with TIMP2 having the highest relative number in tubers (*P* < 0.05; Table 2).

TIMP3 showed a moderate protein expression in neurons in control tissue, while glial cells showed no expression of TIMP3 (Figure 3 **G**,**H**). Strong TIMP3 expression was observed in dysmorphic neurons and moderate expression in giant cells of cortical tubers (Figure 3 **I**). Both the OD and relative number of positive cells are higher in tubers (*P* < 0.01) and perituberal tissue (*P* < 0.05, *P* < 0.01 respectively) compared to controls (Table 2).

Protein expression of TIMP4 was not present in neurons of control cortex; however, moderate expression was seen in glial cells (Figure 3 **J**,**K**). Strong TIMP4 expression was observed in dysmorphic neurons and giant cells of cortical tubers (Figure 3 **L**). The relative number of TIMP4‐positive glia was higher within tubers and perituberal cortex as compared to controls (*P* < 0.01) with glia in tubers displaying the highest relative number (*P* < 0.05; Table 2).

Fluorescent double‐labelling showed co‐localization of all TIMPs with GFAP (Figure 3 insets c), while co‐localization with microglia markers was not evident.

To study whether specific MMPs and TIMPs co‐localize, double‐labelling was performed. Co‐localization of MMP2 and TIMP2 was observed in giant cells and dysmorphic neurons while many glial cells were only immunopositive for MMP2 (Figure S1 **A**). MMP3 co‐localized with TIMP1, while co‐localization with TIMP3 was only seen in some giant cells, but not in glia (Figure S1 **B**,**C**). Co‐localization of MMP9 and TIMP1, as well as MMP14 with TIMP2 and TIMP4 was observed in the majority of cells (Figure S1 **D–F**).

### Increased albumin and CD163 immunoreactivity in cortical tubers

**Figure 4:**
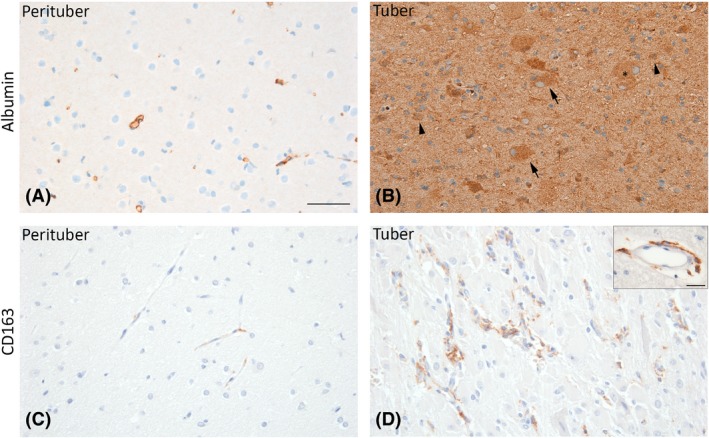
Albumin and CD163 immunoreactivity in tuberous sclerosis complex (TSC) tubers. Albumin extravasation was not observed in perituberal brain tissue of TSC patients (**A**). In tubers, the parenchyma was strongly positive for albumin. Albumin staining was also seen in glial cells, dysmorphic neurons and giant cells within the tuber (**B**). CD163‐positive perivascular macrophages were sparsely present around vessels in perituberal brain tissue (**C**). In tubers, CD163‐positive cells were more frequently observed around blood vessels (**D**) and had an activated morphology with large cell bodies and coarse processes (inset). Arrows depict dysmorphic neurons, arrowheads depict glial cells and asterisks depict giant cells. Scale bar **A**–**D**: 200 μm, scale bar inset: 25 μm.

To assess BBB dysfunction, extravasation of albumin and the presence of CD163‐positive perivascular macrophages were investigated in tubers and the adjacent tissue of TSC patients using immunohistochemistry. In perituberal cortex, albumin extravasation was not observed (Figure 4 **A**). In tubers, however, strong albumin immunoreactivity was seen in the parenchyma. Additionally, astrocytes, dysmorphic neurons and giant cells were albumin‐positive (Figure 4 **B**). CD163‐positive cells were sparsely present around vessels in the perituberal cortex (Figure 4 **C**), while considerably more CD163‐positive cells were observed in tubers. Moreover, CD163‐positive cells in tubers had the morphology of activated macrophages with large cell bodies and coarse processes (Figure 4 **D** inset).

### Modulation of MMPs and TIMPs by miR146a and miR147b in tuber‐derived cultures

**Figure 5:**
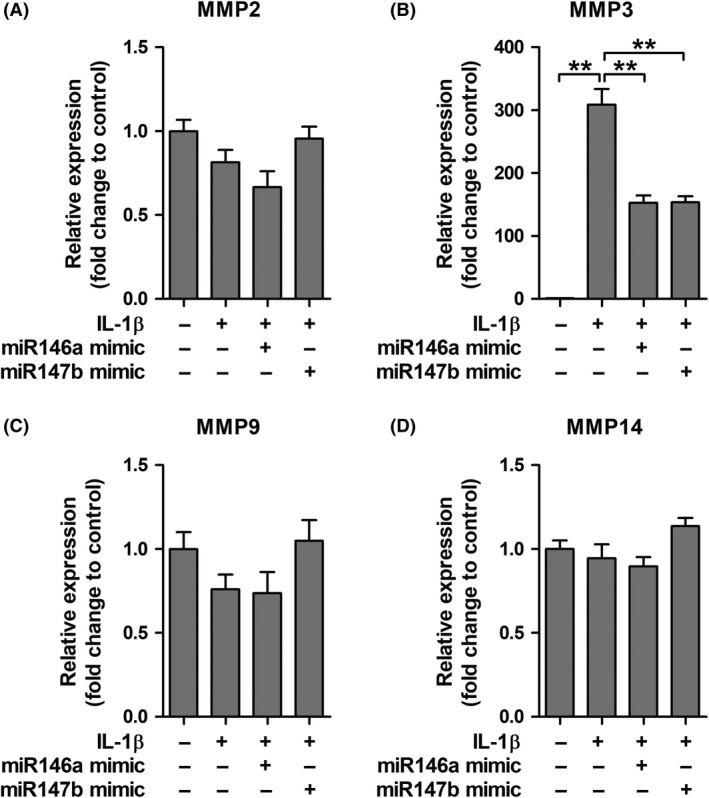
Effects of interleukin (IL)‐1β stimulation and microRNA transfection on matrix metalloproteinases (MMP) expression. Quantitative real‐time PCR of *MMP2*,* MMP3*,* MMP9* and *MMP14* in tuberous sclerosis complex (TSC) tuber‐derived cell cultures. (**B**) Stimulation of cells with IL‐1β led to higher *MMP3* expression, which was attenuated by transfection with either miR146a or miR147b. (**A**,** C**,** D**) The expression of *MMP2*,* MMP9* and *MMP14* did not change after stimulation and/or transfection with microRNAs. **P < 0.01; Mann–Whitney *U*‐Test.

**Figure 6:**
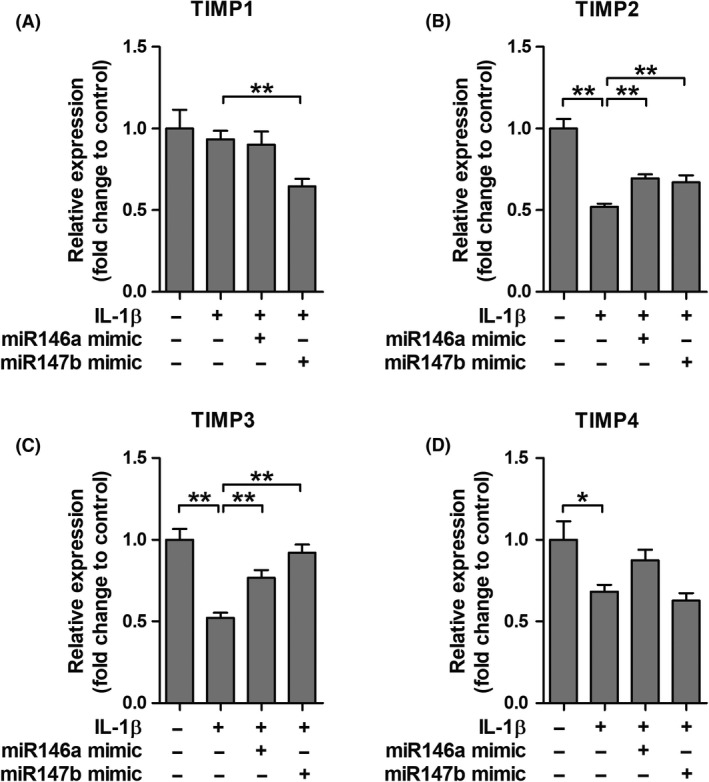
Effects of interleukin (IL)‐1β stimulation and microRNA transfection on tissue inhibitor of metalloproteinase (TIMPs) expression. Quantitative real‐time PCR of *TIMP1*,* TIMP2*,* TIMP3* and *TIMP4* in tuberous sclerosis complex (TSC) tuber‐derived cell cultures. (**B**,**C**,**D**) Stimulation of cells with IL‐1β led to higher *TIMP2*,* TIMP3* and *TIMP4* expression. Decreased *TIMP2* and *TIMP3* mRNA expression could be attenuated by transfection with both miR146a and miR147b. *P < 0.05, **P < 0.01; Mann–Whitney *U*‐Test.

To investigate whether the expression of MMPs and TIMPs could be modulated, TSC tuber‐derived cell cultures were transfected with either miR146a or miR147b mimic molecules and subsequently stimulated with IL‐1β to mimic an inflammatory response. After transfection, Taqman PCRs confirmed the overexpression of miR146a and miR147b 34 indicating successful transfection. *MMP3* expression was higher after IL‐1β stimulation compared to control (*P* = 0.002; Figure 5 **B**), while *MMP2*,* MMP9* and *MMP14* expression did not change (Figure 5 **A**,**C**,**D**). IL‐1β‐induced upregulation of *MMP3* could be attenuated by approximately 50% after transfection with miR146a or miR147b (*P* = 0.002). Following IL‐1β stimulation, expression of *TIMP2*,* TIMP3* and *TIMP4* was lower compared to control (*P* = 0.002, *P* = 0.002, *P* = 0.026 respectively; Figure 6 **B**–**D**). Decreased *TIMP2* and *TIMP3* expression could be attenuated by both miR146a (*P *= 0.002) and miR147b (*P* = 0.009) transfection.

## Discussion

This study provides insight into the dysregulated MMP/TIMP proteolytic system in TSC brain. We demonstrated higher expression of several MMPs and TIMPs in TSC tubers as compared to normal neocortical brain tissue and perituberal cortex, particularly in dysmorphic neurons and giant cells, as well as in glial cells, which was associated with BBB dysfunction. Furthermore, IL‐1β‐induced upregulation of *MMP3* and downregulation of *TIMP2* and *TIMP3* in tuber‐derived cultures could be attenuated by miR146a or miR147b transfection. These findings will be discussed in further detail in the following paragraphs.

### Dysregulation of MMP and TIMP in cortical tubers

We observed that besides higher mRNA expression of *MMP9*,* MMP14* and *TIMP4*, protein expression of MMP2, 3, 9 and 14 and TIMP1, 2, 3 and 4 is abundantly present in cortical tubers of patients with TSC and that particularly glial cells, dysmorphic neurons and giant cells were strongly immunopositive for MMPs. To the best of our knowledge, this is the first study providing a complete picture of the expression and cellular distribution of major brain MMPs and TIMPs in resected tubers of TSC patients. The discrepancy between mRNA and protein expression of MMPs/TIMPs may be attributed to the fact that mRNA data were obtained using homogenized cortical specimens, while protein expression was studied at the cellular level using immunohistochemistry which provides a better resolution as compared to the use of homogenates.

The importance of ECM alterations in the brain of TSC patients has been suggested by sequencing studies investigating the transcriptional landscape of cortical tubers. It has been shown that the ECM was among the most significantly changed GO terms related to increased gene expression in cortical tubers 42. Moreover, out of all 438 differentially expressed genes in TSC tubers found by high‐throughput RNA sequencing, the majority of the elevated genes was associated with ECM organization along with the innate immune response 43. Only in few studies, the expression of a limited number of MMPs was investigated in resected brain tissue of patients with epilepsy‐related disorders. Li and colleagues studied MMP9 expression in TSC and focal cortical dysplasia (FCD), another malformation of cortical development and a well‐known cause of pharmacoresistant epilepsy. They found that MMP9 protein expression was higher in TSC and FCD brain compared to controls and observed high expression of MMP9 in giant cells and dysmorphic neurons 44. Konopka *et al*. observed strong MMP9 expression in dysmorphic neurons and balloon cells in the cortex of FCD patients and showed that neuronal protein expression of MMP9 was higher in FCD patients compared to control brain 45. Besides MMP9, they showed that MMP2, MMP3 and TIMP2 expressions were higher in the dysplastic cortex of adult FCD patients *vs*. controls. In hippocampi of patients with temporal lobe epilepsy (TLE), MMP9 expression was higher compared to controls 44. Furthermore, higher MMP2 and MMP9 activity was found in the epileptogenic and perilesional area of patients with TLE 46. Only one study investigated TIMP expression in a human epilepsy‐related pathology. Acar and colleagues observed pronounced protein expression of TIMP1 and TIMP2 in a small number of TLE patients 47. In the present study, co‐localization of several MMPs and TIMPs was found in glial cells as well as in tuberous cells. However, MMP3 was not found to be co‐localized with TIMP3 in glia and a similar pattern was seen for some, but not all, MMP2‐positive glial cells. Specific MMP and TIMP combinations were chosen based on the interaction between MMPs and TIMPs as described in literature 35. The fact that MMPs and TIMPs can be produced by the same cells and that their expressions are regulated by these cells can have important functional implications. As TIMPs are generally thought to be MMP inhibitors 5, the higher expression of TIMPs in tubers brain may occur in response to higher MMP expression, as a compensatory mechanism. Furthermore, TIMPs may protect the BBB 48. However, knowledge about the precise function and spatio‐temporal expression patterns of MMPs and TIMPs is still somewhat limited and it should be noted that some TIMPs might even be involved in enhancing MMP activity. Controversially, TIMP2, an effective inhibitor of all MMPs, is also responsible for the activation of pro‐MMP2 with the use of two MMP14 molecules 49, 50, leading to cleavage of the propeptide which activates MMP2. Furthermore, TIMP2, TIMP3 and TIMP4 are able to interact with propeptide of MMP2 and TIMP1 and TIMP3 is able to interact with pro‐MMP9 51. Future studies, in which the cellular distribution and expression in mutant animals are taken into account, may provide more answers.

Although the mechanisms underlying the regulation of MMPs and TIMPs in TSC tubers are still unclear, a potential link with the mTOR pathway has been suggested, as both MMPs, TIMPs and mTOR are downstream targets of the PI3K/Akt pathway 52, 53, 54, 55. A recent study showed that inhibition of the mTOR pathway by rapamycin leads to the downregulation of MMP9 activity in models of Alzheimer's disease and vascular cognitive impairment 56. Given the increased activation of the mTOR pathway due to *TSC1/TSC2* mutation, the link between mTOR and MMPs deserves further investigation in relation to cortical tubers.

In the present study, a dysregulation of MMPs and TIMPs was associated with BBB dysfunction, which was shown by abundant albumin extravasation and the presence of CD163‐positive perivascular macrophages in cortical tubers. Increased expression and activation of MMPs are often observed in pathology where the BBB is compromised, such as cerebral ischaemia 57, 58 and traumatic brain injury 59. Indeed, studies using animal models of stroke have shown increased MMP expression and activity along with BBB dysfunction 60. In our previous studies, BBB dysfunction was also reported in both cortical tubers as well as in subependymal giant cell astrocytomas of TSC patients 28, 30.

The basement membrane of brain blood vessels consists of ECM secreted by the surrounding cells and plays an important role in vascular integrity. MMPs are able to degrade several components of this basement membrane such as laminin, fibronectin, heparan sulphate and type IV collagen 61, 62, 63, thereby influencing barrier permeability. Furthermore, several MMPs target tight junction and adherens junction proteins that control cell‐cell and cell‐matrix interactions at the barrier 64. Yang *et al*. showed that MMP2, which is bound and activated by MMP14, targets claudin‐5 and occludin and that fragmentation of these tight junction proteins can be prevented by inhibiting a broad‐spectrum of MMPs 65. As a consequence of basal lamina and tight junction degradation, BBB permeability increases which can allow the extravasation of leucocytes. Interestingly, leucocytes are an important source of MMP9 66, 67 and extravasation can lead to increased MMP2 activation, thereby providing a positive feedback loop on proteolysis 68. Furthermore, by opening the BBB, neuroinflammation can lead to increased expression and activity of MMPs 69, 70. In the present study, we showed that abundant perivascular macrophages were present in the TSC brain. We previously showed that the number of perivascular macrophages is positively correlated with BBB permeability 37. Furthermore, glial cells such as microglia and astrocytes, often with reactive morphology, had higher expression of MMPs in the TSC brain as compared to autopsy control or perituberal brain tissue. In turn, MMPs can activate cytokines such as tumour necrosis factor‐α, IL‐1β and IL‐8 by cleavage of the propeptide 71, 72, 73. Recently, it was shown that besides chemokines and cytokines, cytokine‐induced activity of MMP2 and MMP9 at the BBB also result in increased leucocyte infiltration 74. These studies, together with our observations, suggest that increased MMP activation, BBB disruption and increased neuroinflammation coincide and are able to enhance one another.

Taken together, we showed that dysregulation of MMPs/TIMPs and BBB dysfunction specifically occurs in epileptogenic tubers of TSC patients with recurrent seizures, but not in perilesional tissue. This indicates that, rather than just being a consequence of seizure activity, MMPs may play a crucial role in epilepsy pathology.

One of the main characteristics of TSC tubers is the presence of brain inflammation, in particular a prominent activation of the IL‐1β signalling pathway 28, 42. Several miRNAs have been shown to be important during inflammatory processes in epilepsy‐associated pathologies 75, 76, 77, 78 and have been proposed to have beneficial effects on MMP expression in *in vitro* studies 33. Furthermore, a recent meta‐analysis has shown the involvement of differentially expressed miRNAs in both human and experimental TLE in pathways of inflammation, gliosis and ECM dysregulation 79.

We wondered whether MMP expression could be modulated *in vitro* by anti‐inflammatory miR146a or miR147b. Stimulation of tuber‐derived cells with IL‐1β resulted in higher mRNA expression of *MMP3*, though it did not affect the mRNA expression of *MMP2*,* MMP9* and *MMP14*. This might be explained by the fact that the cells, taken from the cortical tubers of TSC patients, already exhibit a high amount of MMP2, MMP9 and MMP14 transcripts and are therefore not sensitive to further induction by pro‐inflammatory molecules. Moreover, mRNA expression of *TIMP2*,* TIMP3* and *TIMP4* was lower after IL‐1β stimulation compared to controls, indicating a misbalance in the MMP/TIMP proteolytic system after this inflammatory stimulus. We found that IL‐1β‐induced upregulation of *MMP3* and downregulation of *TIMPs* could be attenuated by miR146a or miR147b in tuber‐derived cell cultures, thereby re‐establishing the balance. Previously, we have shown that inhibition of pro‐inflammatory miR155 attenuated IL‐1β‐induced *MMP3* overexpression in human foetal astrocytes 33. miR146a has been shown to inhibit MMP2 expression in prostate cancer tissue 80 and the activity of MMP9 in human cardiac cells 81. In breast cancer cells, miR146a negatively regulates the activity of NFκB, thereby suppressing the expression of NFκB target gene *MMP9* 82. Furthermore, it was shown that transient treatment with miR146a mimic molecules after kainic acid‐induced epilepsy in mice had disease‐modifying effects as it decreased seizure recurrence and prevented disease progression 83. As it has been shown that both miR146a and miR147b are negative‐feedback regulators of astrocyte‐mediated inflammatory response 34, 77, 84, it is tempting to speculate that these miRNAs inhibit MMP expression in pathological tissue, but this needs to be further investigated.

This study shows that the expression of MMPs and TIMPs is dysregulated and associated with BBB dysfunction in cortical tubers which may contribute to the pathological brain networks in TSC. Dysregulated MMP and TIMP expression can be ameliorated *in vitro* by the anti‐inflammatory miR146a or miR147b. Thus, these miRNAs deserve further investigation as a novel therapeutic approach to restore the BBB and the ECM.

## Author contributions

DWMB, JvS, EA and EAvV conceived and designed the experiments. FEJ, WGS, PCvR have provided human brain tissue which was analysed and diagnosed by EA. DWMB, JvS and JJA processed human brain tissue. DWMB performed immunohistochemistry and analysed the data. DWMB and JvS performed RT‐qPCR and analysed the data. DWMB, JvS, LW, BvhH, WWK and HEdV were involved in the *in vitro* experiments. DWMB, JvS, EA and EAvV contributed to the data interpretation and writing of the manuscript. All authors read, revised and approved the final manuscript.

## Disclosure

The authors have no conflicts of interest to report. We confirm that we have read the Journal's position on issues involved in ethical publication and affirm that this report is consistent with those guidelines.

## Supporting information


**Figure S1.** Co‐localization of matrix metalloproteinases (MMPs) and tissue inhibitor of metalloproteinase (TIMPs) in cortical tubers. Co‐localization of MMP2 and TIMP2 was observed in giant cells and dysmorphic neurons, while glial cells were only MMP2‐positive (**A**). MMP3 co‐localized with TIMP1 in all cells and with TIMP3 in some giant cells, but not in glia (**B**,**C**). Co‐localization was observed between MMP9 and TIMP1 (**D**) and MMP14 with both TIMP2 and TIMP4 (**E**,**F**). Arrowheads depict glial cells and asterisk depict giant cell. Scale bar **A**–**F**: 25 μm.

**Table S1.** Clinical findings of patients with tuberous sclerosis complex (TSC) (1–20) and autopsy controls (21–43).
