# Supplementary figures and images for: Increased matrix metalloproteinases expression in tuberous sclerosis complex: modulation by microRNA 146a and 147b in vitro
Source: Neuropathol Appl Neurobiol. 2019 Jul 1;46(2):142–59. doi: 10.1111/nan.12572 (PMC7217197; doi:10.1111/nan.12572)

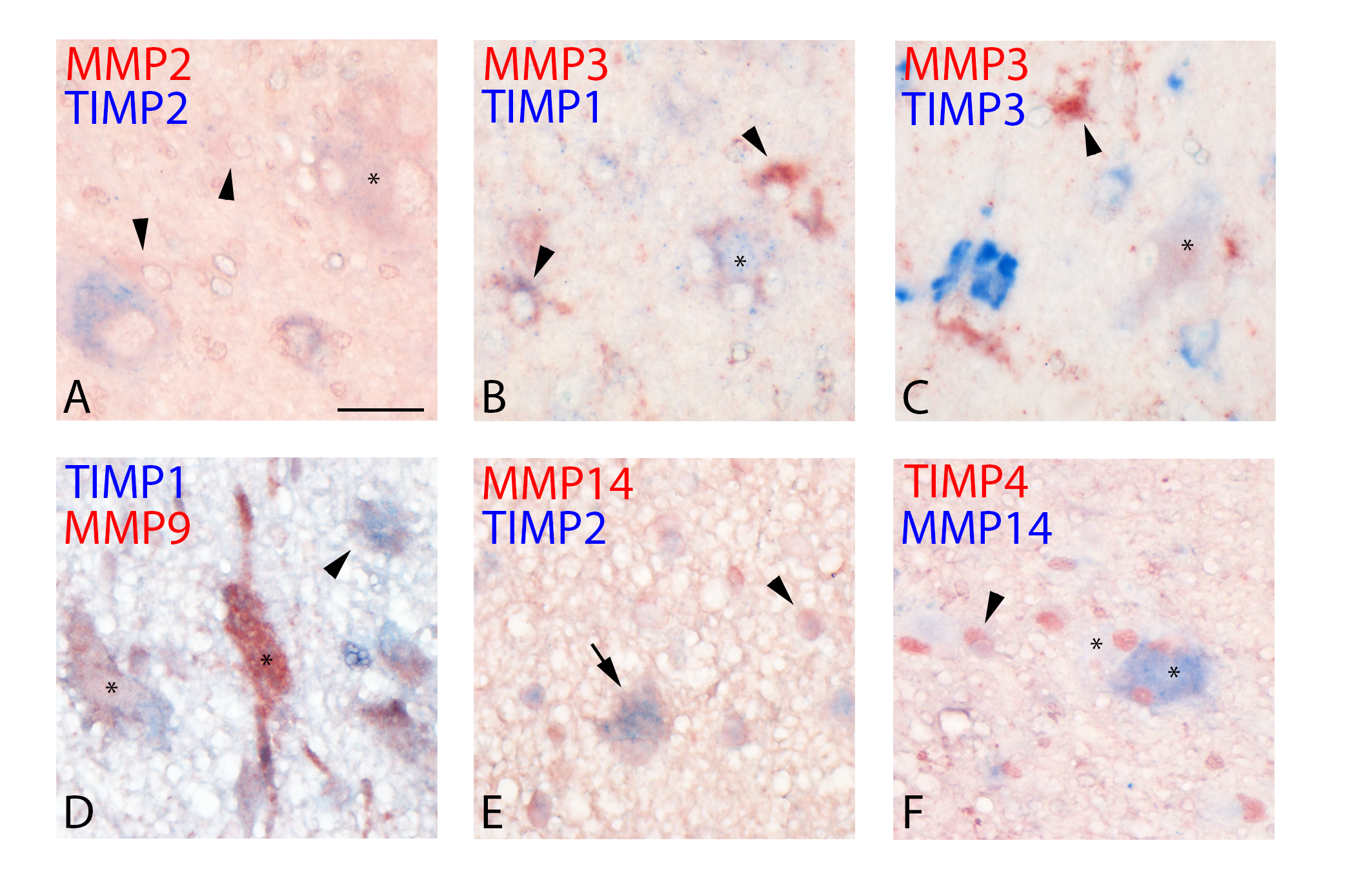

Supplement: Supplementary file 1 — Figure S1. Co‐localization of matrix metalloproteinases (MMPs) and tissue inhibitor of metalloproteinase (TIMPs) in cortical tubers. Co‐localization of MMP2 and TIMP2 was observed in giant cells and dysmorphic neurons, while glial cells were only MMP2‐positive (A). MMP3 co‐localized with TIMP1 in all cells and with TIMP3 in some giant cells, but not in glia (B,C). Co‐localization was observed between MMP9 and TIMP1 (D) and MMP14 with both TIMP2 and TIMP4 (E,F). Arrowheads depict glial cells and asterisk depict giant cell. Scale bar A–F: 25 μm. [file NAN-46-142-s001.tif]
