# Supplementary material for: Increased matrix metalloproteinases expression in tuberous sclerosis complex: modulation by microRNA 146a and 147b in vitro
Source: Neuropathol Appl Neurobiol. 2019 Jul 1;46(2):142–59. doi: 10.1111/nan.12572 (PMC7217197; doi:10.1111/nan.12572)
Supplement: Supplementary file 2 — Table S1. Clinical findings of patients with tuberous sclerosis complex (TSC) (1–20) and autopsy controls (21–43). [file NAN-46-142-s002.docx]

**Supplementary Table 1.** Clinical findings of patients with TSC (1-20) and autopsy controls (21-43)

| **Patient** | | **Gender** | **Localization** | **Age of surgery** | **Mutation** | **Duration of** | **Seizure frequency** |
| --- | --- | --- | --- | --- | --- | --- | --- |
|  |  |  |  | **(year)** |  | **epilepsy (year)** | **(per month)** |
| **1** | **a** | m | T | 16 | TSC2 | 13 | 1 |
| **2** | **a** | f | F | 4 | TSC1 | 1.333 | 30.5 |
| **3** | **a** | m | F | 2 | TSC2 | 1.75 | 30.5 |
| **4** | **a,b** | f | F | 13 | TSC2 | 13 | 91.5 |
| **5** | **a** | f | F | 5 | TSC1 | 3 | 183 |
| **6** | **a,b** | m | P | 3 | TSC2 | 3 | 213.5 |
| **7** | **b** | f | F | 13 | TSC2 | 13 | 91.5 |
| **8** | **b** | m | P | 8 | TSC1 | 8 | 15 |
| **9** | **b** | m | P | 8 | TSC1 | 8 | 15 |
| **10** | **b** | m | F | 32 | TSC2 | 31.8 | 20 |
| **11** | **b** | f | F | 42 | TSC2 | 41 | 25 |
| **12** | **b** | f | F/P | 21 | TSC1 | 1 | 30 |
| **13** | **b** | m | F | 35 | TSC2 | 31 | 25 |
| **14** | **b** | m | T | 0.92 | TSC2 | 0.92 | 200 |
| **15** | **b** | f | P | 10 | TSC2 | 10 | 225 |
| **16** | **b** | m | F | 47 | TSC2 | 35 | 30 |
| **17** | **b** | m | F | 3 | TSC2 | 2.42 | 180 |
| **18** | **b** | m | F | 10 | TSC2 | 8 | 225 |
| **19** | **b** | f | F | 0.83 | NA | 0.81 | 240 |
| **20** | **b** | m | F | 4 | NA | 0.25 | 50 |
| **21** | **a** | f | F | 17 | - | - | - |
| **22** | **a** | f | F | 7 | - | - | - |
| **23** | **a,b** | f | F | 2 | - | - | - |
| **24** | **a,b** | m | F | 13 | - | - | - |
| **25** | **a,b** | m | T | 10 | - | - | - |
| **26** | **a** | m | F | 2 | - | - | - |
| **27** | **b** | f | T | 0.17 | - | - | - |
| **28** | **b** | f | F | 0.17 | - | - | - |
| **29** | **b** | f | F | 2 | - | - | - |
| **30** | **b** | f | T | 0.25 | - | - | - |
| **31** | **b** | m | F | 10 | - | - | - |
| **32** | **b** | f | T | 17 | - | - | - |
| **33** | **b** | f | T | 17 | - | - | - |
| **34** | **b** | f | T | 13 | - | - | - |
| **35** | **b** | f | P | 44 | - | - | - |
| **36** | **b** | f | F | 39 | - | - | - |
| **37** | **b** | f | F | 25 | - | - | - |
| **38** | **b** | m | F | 56 | - | - | - |
| **39** | **b** | m | F | 30 | - | - | - |
| **40** | **b** | m | F | 31 | - | - | - |
| **41** | **b** | f | T | 66 | - | - | - |
| **42** | **b** | m | F | 57 | - | - | - |
| **43** | **b** | m | F | 82 | - | - | - |
| m, male; f, female; F, frontal; P, Parietal; T, temporal; NA, not available | | | | | | | |
| a | used for immunohistochemistry | | | |  |  |  |
| b | used for real-time PCR | | |  |  |  |  |
